# Staphylococcus aureus enterotoxin A- and B-specific IgE in chronic obstructive pulmonary disease

**DOI:** 10.1186/s12931-023-02520-4

**Published:** 2023-09-22

**Authors:** Meropi Karakioulaki, Caroline Maria Berkemeier, Ingmar Heijnen, Leticia Grize, Eleni Papakonstantinou, Antonis Goulas, Michael Tamm, Daiana Stolz

**Affiliations:** 1 Clinic of Respiratory Medicine and Pulmonary Cell Research, University Hospital Basel, Basel, Switzerland; 2 First Laboratory of Pharmacology, School of Medicine, Faculty of Health Sciences, Aristotle University of Thessaloniki, Thessaloniki, Greece; 3 Medical Immunology, Department of Laboratory Medicine, University Hospital Basel, Basel, Switzerland; 4 Clinic of Respiratory Medicine, Faculty of Medicine, Universitätsklinikum Freiburg, Freiburg, Germany; 5 Clinical Research Unit, Special Unit for Biomedical Research and Education, School of Medicine, Aristotle University of Thessaloniki, Thessaloniki, Greece

**Keywords:** COPD, Asthma, Staphylococcus aureus, Staphylococcal enterotoxin A and B, IgE, Allergy, Atopy

## Abstract

Sensitization to Staphylococcus aureus enterotoxins A (SEA) and B (SEB) has been associated with asthma severity, exacerbations, and disease control. Our study aimed to investigate if there are differences in serum SEA-IgE and SEB-IgE levels between patients with chronic obstructive pulmonary disease (COPD), asthma, and controls, and to assess the association between SE sensitization and COPD clinical parameters and Th2 inflammation biomarkers in two well-defined COPD cohorts. Our findings suggest that COPD patients do not exhibit higher SEA and SEB sensitization compared to asthma patients and controls. However, in COPD patients, the presence of atopy and allergy is associated with positivity for SEA-IgE and SEB-IgE. Consequently, these allergens may aid in identifying atopic or allergic subgroups within the COPD population, but they are not directly associated with the diagnosis of COPD, elevated circulating blood eosinophils, or fractional exhaled nitric oxide (FENO) levels.

## Introduction

*Staphylococcous aureus* (*S. aureus*) is a common opportunistic pathogen that colonizes the airways [1]. Among more than 20 staphylococcal enterotoxins, staphylococcal enterotoxin A (SEA) and B (SEB) are the best characterized and defined as superantigens, because they are able to induce a polyclonal activation of T-cells, leading to a cytokine bolus and acute toxic shock [2]. Asthmatic patients have increased specific IgE to various secreted *S. aureus* proteins, and a number of studies have demonstrated that sensitization to staphylococcal enterotoxins (SE) is associated with asthma severity [3–7], asthma exacerbations [4], asthma control and age of asthma onset [8, 9]. So far, there is only one study suggesting that levels of specific IgE to SE (SE-IgE) are higher in both stable and exacerbated COPD subjects when compared to controls [10]. In this study, we aim to elucidate whether there are differences in serum specific IgE to SEA (SEA-IgE) and SEB (SEB-IgE) between COPD, asthma patients, and control individuals, and examine the association of SEA-IgE and/ or SEB-IgE with clinically relevant COPD outcomes.

## Methods

This study consists of two cohorts. The derivation cohort includes 73 COPD patients (EKBB 295/07), 19 severe asthma patients (EKNZ 2016 − 01057) and 27 sex-matched blood donors, smokers without asthma or COPD (control group). The confirmation cohort consisted of 342 well characterized COPD patients, derived from the PREVENT study (ISRCTN 45,572,998, EKBB 306/10).

Clinical, laboratory, lung function, as well as Th2 inflammation characteristics [total IgE levels (tIgE), skin prick test, blood eosinophils, fractional exhaled nitric oxide (FeNO)] were collected from all patients. The ALEX^2^ Allergy Explorer assay (Macro Array Diagnostics, Wien, Austria) was utilized to measure tIgE. Patients were considered atopic when tIgE ≥ 100 kU/L and allergic when their skin prick test was positive, as previously described [11]. Additionally, serum specific SEA-IgE and SEB-IgE were measured using the monoplex ImmunoCAP assay (ThermoFisher Diagnostics, Uppsala Sweden), following the manufacturer’s instructions. Values above > 0.35 kU_A_/L were considered positive.

## Results

In the derivation cohort, SEA-IgE positivity was observed in 4 (5.48%) COPD, 2 (10.53%) asthma and 1 (3.70%) control, whereas SEB-IgE positivity was present in 9 (12.33%), 4 (21.05%) and 4 (14.81%) subjects, respectively. Neither the percentages nor the concentrations of SEA-IgE (p = 0.639 and p = 0.280, respectively) and SEB-IgE (p = 0.609 and p = 0.342, respectively) differed significantly between the various groups. In COPD patients we found no association between markers of Th2 inflammation [blood eosinophils (N = 70), FeNO (N = 47), history of allergy (N = 28)], neither with SEA-IgE (p = 0.779, p = 0.869, and p = 0.750, respectively) nor with SEB-IgE positivity (p = 0.881, p = 0.737, and p = 0.594, respectively).

In the confirmation cohort, there was a significant difference with respect to frequency of SEA-IgE positivity between men and women (p = 0.014, Table 1). SEB-IgE positivity was associated with lower incidence of severe exacerbation as compared to SEB-IgE negativity (20.00% vs. 27.15%, p = 0.006, Table 1). Indeed, SEB-IgE positive subjects had a longer time to first AECOPD than SEB-IgE negative subjects (571.769 days vs. 383.968 days, p = 0.023). Additionally, an increase of 1kUA/L in SEB-IgE prolonged the time to first AECOPD from baseline by 42.995 days (p = 0.009). Allergy, atopy, and positivity to a rhinitis-related skin prick allergen were, as expected, more commonly observed among SEA-IgE and SEB-IgE positive patients (Table 1).

Age, cigarette exposure in pack years, Global Initiative for Chronic Obstructive Lung Disease grade, post bronchodilator body plethysmography values, walking distance in the 6-Minute Walking Test, health related life quality as assessed by the St. George Respiratory Questionnaire, symptoms as assessed by the COPD assessment test and Modified Medical Research Council Dyspnea Scale scores, number and etiology of AECOPD in the previous year and during the PREVENT study, circulating blood eosinophils and sputum microbiology were similar between SEA-IgE- and SEB-IgE- positive and negative patients (Table 1).

**Table 1:** Comparisons of COPD clinical parameters between patients that were SEA- / SEB- IgE positive and negative in the confirmation cohort of the study (N = 342)

Parameter	SEA-IgE positive	SEA-IgE negative	p-value	SEB-IgE positive	SEB-IgE negative	p-value
**Gender, (female %)**	3 (10.71%), N = 28	104 (33.12%), N = 314	**0.014**	8 (20.00%), N = 40	99 (32.78%), N = 302	0.107
**Age, median (IQR)**	69.41 (58.00-76.24) N = 28	67.25 (62.04–73.82) N = 314	0.607	67.21 (56.67–76.36) N = 40	67.33 (62.23–73.82) N = 302	0.526
**PY, median (IQR)**	47.50 (30.00-78.75) N = 28	50.00 (35.00–70.00) N = 314	0.681	50.00 (40.00-63.75) N = 40	50.00 (30.00–70.00) N = 302	0.651
**Current smoker, N (%)**	12 (42.86%), N = 28	112 (35.67%), N = 314	0.448	20 (50.00%), N = 40	104 (34.44%), N = 302	0.054
**GOLD Grade, N (%)**						
GOLD 1	0	13 (4.35%), N = 299	0.714	2 (5.13%), N = 39	11 (3.82%), N = 288	0.813
GOLD 2	17 (60.72%), N = 28	174 (58.19%), N = 299		20 (51.28%), N = 39	171 (59.38%), N = 288	
GOLD 3	8 (28.57%), N = 28	86 (28.76%), N = 299		13 (33.33%), N = 39	81 (28.13%), N = 288	
GOLD 4	3 (10.71%), N = 28	26 (8.70%), N = 299		4 (10.26%), N = 39	25 (8.68%), N = 288	
**Post bronchodilator body plethysmography**						
FVC, median (IQR)	3.31 (2.62–4.01), N = 23	3.20 (2.65–3.86), N = 263	0.847	3.56 (2.81–4.16), N = 34	3.19 (2.64–3.85), N = 252	0.307
FVC %, median (IQR)	83.40 (69.40-94.78) N = 28	86.50 (74.70-100.50) N = 299	0.250	86.50 (77.10–99.20) N = 39	86.30 (74.35-100.48) N = 288	0.904
FEV_1_, median (IQR)	1.44 (0.95–1.83), N = 28	1.43 (1.03–1.84), N = 299	0.947	1.44 (0.96–2.15), N = 39	1.43 (1.04–1.83), N = 288	0.517
FEV_1_%, median (IQR)	57.85 (39.65–65.55) N = 28	56.00 (43.50–67.80) N = 299	0.711	55.00 (43.10–67.80) N = 39	56.05 (43.28–67.78) N = 288	0.675
RV%, median (IQR)	139.00 (118.63-175.43) N = 24	143.10 (122.10-170.60) N = 263	0.818	143.80 (116.65–177.00), N = 34	142.50 (122.35–169.80) N = 253	0.879
TLC, median (IQR)	6.90 (5.89–7.97), N = 24	6.68 (5.62–7.66), N = 263	0.421	6.88 (6.01–8.21), N = 34	6.68 (5.62–7.66), N = 253	0.253
TLC%, median (IQR)	111.80 (100.00-123.75) N = 24	112.10 (98.90–127.00) N = 263	0.843	109.40 (100.00-129.73), N = 34	112.30 (98.75-126.35) N = 253	0.968
RV%TLC%, median (IQR)	50.47 (42.54–61.11) N = 24	50.06 (44.11–55.86) N = 262	0.886	50.73 (41.40-57.59) N = 34	49.99 (44.19–55.67) N = 252	0.918
DLCO SB%, median (IQR)	55.10 (42.35-71.00) N = 24	56.30 (43.20–69.00) N = 259	0.962	55.95 (41.18–72.45) N = 36	55.90 (43.20–69.00) N = 247	0.853
DLCOc SB%, median (IQR)	63.45 (45.13–79.03) N = 12	54.25 (37.48–67.33) N = 108	0.119	63.20 (46.25–74.60) N = 13	53.90 (37.30–67.40) N = 107	0.112
DLCO/VA%, median (IQR)	67.45 (53.55–87.78) N = 24	66.90 (52.50-87.85) N = 257	0.941	64.40 (52.58–87.20) N = 36	67.90 (52.80-87.85) N = 245	0.596
DLCOc/VA%, median (IQR)	76.65 (62.18-102.15) N = 12	65.40 (44.18–87.23) N = 108	0.109	63.20 (53.32–85.60) N = 14	66.90 (45.13–91.28) N = 106	0.941
**FeNO (ppm), median (IQR)**	19.50 (10.00-36.50)	16.50 (11.00-24.50)	0.570	14.50 (11.00–27.00)	16.50 (11.00–26.00)	0.620
**6MWT** distance walked in 6-minutes, median (IQR)	412.00 (360.00-485.00) Ν = 21	399.00 (320.00-472.75) Ν = 282	0.536	390.00 (306.00-480.00) Ν = 35	400.00 (321.75-470.75) Ν = 268	0.664
**SGRQ score, median (IQR)**	30.62 (20.50-40.93) N = 26	35.28 (24.65–49.87) N = 308	0.189	32.32 (19.77–45.09) N = 39	34.69 (24.68–49.97) N = 295	0.289
**CAT score, median (IQR)**	12.00 (7.00–19.00) N = 27	14.00 (10.00-19.50) N = 313	0.191	14.00 (8.00–20.00) N = 39	14.00 (10.00–19.00) N = 301	0.512
**MMRC score, N (%)**						
1	8 (29.63%), N = 27	50 (16.29%), N = 307	0.392	13 (32.50%), N = 40	45 (15.31%), N = 294	0.080
2	6 (22.22%), N = 27	101 (32.90%), N = 307		8 (20.00%), N = 40	99 (33.67%), N = 294	
3	10 (37.04%), N = 27	110 (35.83%), N = 307		14 (35.00%), N = 40	106 (36.05%), N = 294	
4	3 (11.11%), N = 27	39 (12.70%), N = 307		4 (10.00%), N = 40	38 (12.93%), N = 294	
5	0, N = 27	7 (2.28%), N = 307		1 (2.50%), N = 40	6 (2.04%), N = 294	
**Symptoms at baseline, N (%)**						
Shortness of breath	11 (39.29%), N = 28	121 (38.54%), N = 314	0.938	12 (30.00%), N = 40	120 (39.74%), N = 302	0.235
Cough	13 (46.43%), N = 28	97 (30.89%), N = 314	0.092	16 (40.00%), N = 40	94 (31.13%), N = 302	0.259
Wheezing	2 (7.14%), N = 28	25 (7.96%), N = 314	0.878	5 (12.50%), N = 40	22 (7.29%), N = 302	0.250
Sputum production	14 (50.00%), N = 28	116 (36.94%), N = 314	0.173	13 (32.59%), N = 40	117 (38.74%), N = 302	0.445
**Unscheduled Urgent Physician Visits due to AECOPD in the previous year, N (%)**	23 (82.14%), N = 28	280 (89.17%), N = 314	0.262	34 (85.00%), N = 40	269 (89.07%), N = 302	0.446
**Severe AECOPD in the previous year, N (%)**						
0	24 (85.71%), N = 28	228 (72.61%), N = 314	0.272	32 (80.00%), N = 40	220 (72.85%), N = 302	**0.006**
1	4 (14.29%), N = 28	75 (23.89%), N = 314		4 (10.00%), N = 40	75 (24.83%), N = 302	
2	0	11 (3.50%), N = 314		4 (10.00%), N = 40	7 (2.32%), N = 302	
**Bacterial AECOPD in the previous year, N (%)**	14 (50.00%), N = 28	161 (51.27%), Ν = 314	0.897	17 (42.50%), Ν = 40	158 (52.32%), N = 302	0.243
**Eosinophilic AECOPD in the previous year, N (%)**	9 (32.14%), Ν = 28	121 (38.53%), N = 314	0.504	15 (37.50%), N = 40	115 (38.08%), N = 302	0.943
**Total number of AECOPD during the PREVENT study, N (%)**						
0	20 (71.43%), N = 28	215 (68.47%), N = 314	0.965	27 (67.50%), N = 40	208 (68.87%), N = 302	0.965
1	6 (21.43%), N = 28	64 (20.38%), N = 314		10 (25.00%), N = 40	60 (19.87%), N = 302	
2	2 (7.14%), N = 28	20 (6.37%), N = 314		2 (5.00%), N = 40	20 (6.62%), N = 302	
3	0	9 (2.87%), N = 314		1 (2.50%), N = 40	8 (2.65%), N = 302	
4	0	2 (0.64%), N = 314		0	2 (0.66%), N = 302	
5	0	3 (0.96%), N = 314		0	3 (0.99%), N = 302	
7	0	1 (0.32%), N = 314		0	1 (0.33%), N = 302	
**Type of AECOPD during the PREVENT study, N (%)**						
Viral only, Ν = 53	3 (5.66%)	50 (94.34%)	0.433	4 (7.55%)	49 (92.45%)	0.617
Bacterial only, N = 20	3 (15.00%)	17 (85.00%)		3 (15.00%)	17 (85.00%)	
Viral and Bacterial Concomitant, N = 12	1 (8.33%)	11 (91.67%)		1 (8.33%)	11 (91.67%)	
**Eosinophils in blood X10** ^**9**^ **cells/l, median (IQR)**	0.15 (0.11–0.31)	0.16 (0.10–0.25)	0.742	0.14 (0.10–0.22)	0.17 (0.10–0.27)	0.356
**Skin prick test positive (allergic), N (%)**	11 (40.74%), N = 27	73 (23.55%), N = 310	**0.048**	15 (38.46%), N = 39	69 (23.15%), N = 298	**0.038**
**Positive to at least one rhinitis related allergen, N (%)**	9 (33.33%), N = 27	54 (17.42%), N = 310	**0.042**	13 (33.33%), N = 39	50 (16.78%), N = 298	**0.013**
**Positive to at least one asthma related allergen, N (%)**	5 (18.52%), N = 27	47 (15.16%), N = 310	0.643	6 (15.38%), N = 39	46 (15.44%), N = 298	0.993
**Atopy (tIgE > 100 kU/L), N (%)**	19 (67.86%), N = 28	47 (14.97%), N = 314	**< 0.001**	27 (67.50%), Ν = 40	39 (12.91%), N = 302	**< 0.001**

**Anti-SEA IgE**: anti-staphylococcal enterotoxin A immunoglobulin E, **anti-SEB IgE**: anti-staphylococcal enterotoxin B immunoglobulin E, **COPD**: Chronic Obstructive Pulmonary Disease, **IQR**: Interquartile Range, **PY**: Pack years, **SGRQ**: St. George Respiratory Questionnaire, **CAT**: COPD assessment test, **FVC**: Forced Vital Capacity, **FEV**_**1**_: Forced expiratory Volume during the first second, **RV**: residual volume, **TLC**: total lung capacity, **DLCO**: Diffusing capacity of lungs for carbon monoxide, **DLCOc**: hemoglobin adjusted DLCO, **SB**: single breath, **VA**: alveolar volume, **FeNO**: fractional nitric oxide concentration in exhaled breath, **MMRC**: Modified Medical Research Council Dyspnea Scale, **6ΜWT**: 6-Minute Walking Test, **GOLD**: Global Initiative for Chronic Obstructive Lung Disease, **AECOPD**: Acute exacerbations of COPD. Comparisons of numeric variables were conducted utilizing the Mann-Whitney U test, while comparisons of categorical variables were conducted utilizing the Pearson’s chi-squared test.

.

## Discussion

This study has demonstrated a similar prevalence of SEA and SEB sensitization among asthma, COPD and controls. SEB-IgE positivity is more commonly encountered than SEA-IgE positivity among COPD patients. A previous study including 18 stable and 54 exacerbated COPD patients reported that SE-IgE positivity is 4 times more frequent in COPD patients compared to healthy controls [10]. However, in contrast to the current study, specific IgE was determined for a mix of SE, including SEA, SEC and TSST-1 [10]. Additionally, our study reveals that a higher proportion of SEB-IgE-positive patients, compared to SEB-IgE-negative patients, remained free of severe AECOPD. In accordance, we found that SEB-IgE positivity, as well as increase in SEB-IgE concentration were associated with a longer time to first AECOPD from baseline. Thus, these findings refute the hypothesis that SEB sensitization is linked to a higher risk of AECOPD. It is worth noting that, to the best of our knowledge, this is the first study ever examining the association across SEB-IgE positivity and SEB-IgE levels and the risk and time to AECOPD. The cause of this association remains so far, however, a conundrum. A previous study including a selected population of 77 patients with COPD suggested that specific IgE to perennial allergens could be linked to selected negative COPD outcomes such as emergency department visit and/or hospitalization, but not steroid use or pneumonia. Unfortunately, sensitization to SAB or SEB has not been evaluated in that study [12].

*S. aureus* is frequently found colonizing patients with Th2-biased diseases, such as atopic dermatitis and chronic rhinosinusitis with nasal polyps. In these patients, there is evidence that *S. aureus* colonizes the nasal mucosa, enclosed in a biofilm or hiding inside immune cells, and constantly produces a panel of factors that could initiate and aggravate Th2-biased immune responses as immune escape mechanisms [13, 14]. Moreover, SE may damage epithelial layers and thus facilitate allergen entry, therefore promoting Th2 airway inflammation [15]. Indeed, our results indicate that in COPD, more SEA-IgE and SEB-IgE positive patients were allergic, atopic, or positive to at least one rhinitis-related skin prick test allergen, when compared to SEA-IgE and SEB-IgE negative patients. In contrast, we found no association with SEA-IgE or SEB-IgE positivity with other Th2 inflammatory markers (such as FeNO or blood eosinophils) among COPD patients of both arms of the study.

IgE with specificity for staphylococcal antigens demonstrates exposure of the immune system to staphylococcal products and does not necessarily indicate presence of *S. aureus* in the airways. *S. aureus*-derived extracellular vesicles are present in the environment and specific staphylococcal proteins (SEA, SEB, SEC) have been detected in dust samples from houses of asthmatic patients [16, 17]. *S.aureus* may even be part of the microbiome of house dust mites found in dust samples [16, 18]. However, it would be of great interest to investigate whether there is an association between the presence of *S. aureus* in the airways of asthmatic and COPD patients and SEA-IgE or SEB-IgE positivity, as relative studies are lacking.

The relatively small sample size of the derivation cohort, which includes populations of unequal size, is a limitation of our study and larger trials with balanced groups are needed to further validate the results. Another limitation of the study can be considered the absence of a confirmation cohort for patients with asthma that would confirm the results obtained in the derivation cohort.

## Concluding remarks

This is, to the best of our knowledge, the first study investigating the association of SEA and SEB sensitization with COPD clinical parameters and Th2 inflammation biomarkers in two well-defined COPD cohorts.

This study demonstrates that although COPD patients do not appear to have a higher frequency of SEA and SEB sensitization than asthma patients and controls, atopy and allergy are both associated with SEA-IgE and SEB-IgE positivity in COPD patients. Thus, these allergens may help to identify atopic or allergic COPD subgroups but cannot be directly associated with the diagnosis of COPD.

## Data Availability

The datasets used and analysed during the current study are available from the corresponding author on reasonable request.

## Abbreviations

FeNO: Fractional exhaled nitric oxide
ICS: Inhaled corticosteroids
LABA: Long-acting beta-agonists
LAMA: Long-acting muscarinic antagonists
*S. aureus*: *Staphylococcous aureus*
SABA: Short-acting beta-agonists
SE-IgE: Specific IgE to SE
SE: Staphylococcal enterotoxins
SEA-IgE: Specific IgE to SEA
SEA: Staphylococcal enterotoxin A
SEB-IgE: Specific IgE to SEB
SEB: Staphylococcal enterotoxin B
SG: Systemic glucocorticosteroids
tIgE: Total IgE levels
